# Demographic and Clinical Findings in Pediatric Patients Affected by Organic Acidemia

**Published:** 2016

**Authors:** Reza NAJAFI, Mahin HASHEMIPOUR, Neda MOSTOFIZADEH, Mohammadreza GHAZAVI, Jafar NASIRI, Armindokht SHAHSANAI, Fatemeh FAMORI, Fatemeh NAJAFI, Mohammad MOAFI

**Affiliations:** 1 Pediatric Endocrinology Department, Ilam University of Medical Sciences, Ilam, Iran; 2 Pediatric Endocrinology Department, Endocrine Research Center, Isfahan University of Medical Sciences, Isfahan, Iran; 3 Pediatric Neurology Department, Isfahan University of Medical Sciences, Isfahan, Iran; 4 Department of Community Medicine, Child Growth and Development Research Center & Research Institute for Primordial Prevention of Non-communicable Disease, Isfahan University of Medical Sciences, Isfahan, Iran; 5 Pediatric gastroenterology Department, Isfahan University of Medical Sciences, Isfahan, Iran; 6 Internal and surgical nursing, Ilam university of medical sciences, Ilam, Iran; 7 Acquired Immunodeficiency Research Center, Isfahan University of Medical Sciences, Isfahan, Iran

**Keywords:** Organic academia, Consanguinity, Developmental delay, Early Detection

## Abstract

**Objective:**

Metabolic disorders, which involve many different organs, can be ascribed to enzyme deficiency or dysfunction and manifest with a wide range of clinical symptoms. This study evaluated some of the demographic and clinical findings in pediatric patients affected by organic acidemia.

**Materials & Methods:**

This cross-sectional study was part of a larger study conducted in patients with metabolic disorders during a period of 7 years from 2007 to 2014 in Isfahan Province, Iran. Our study covered a wide range of cases from newborn infants (one-week old) to adolescents (children up to the age of 17 years). This study evaluated patients’ demographic information, history of disease, developmental and educational status, clinical and general conditions. Phone and in-person interviews were used to gather information.

**Results:**

Out of 5100 patients screened in this study, 392 patients were affected by one of the different metabolic disorders and 167 individuals were diagnosed as organic acidemia. Propionic acidemia/methyl malonic acidemia (PA/MMA) was the most prevalent form of this metabolic disorder. The frequency of consanguinity was 84.7% in the group of patients. The mortality rate was 18.8% in patients with organic academia.

**Conclusion:**

Each of the metabolic diseases, as a separate entity, is rare; nevertheless, in aggregate they have a somewhat high overall prevalence. These diseases result in mental and developmental disorders in the absence of quick diagnosis and initiation of treatment. Furthermore, more mutations should be identified in societies affected by consanguinity. Further research should also be conducted to determine worthwhile and more-efficient screening methods as well as long term neurological prognosis.

## Introduction

Metabolic disorders, ascribable to enzyme deficiency or dysfunction (especially cofactor deficiency), lead to either excess or lack of special metabolic components (1). These inherited metabolic diseases are mostly revealed during infancy or neonatal ages; however, they can occur at any age, even in adulthood. Genetic defects can result in either catabolism or abnormal anabolism of proteins, carbohydrates, lipids, and other complex molecules (2). These diseases are generally attributed to default of enzymes constituting metabolic pathways. Genetic defects lead to accumulation of abnormal biochemical substrates that may potentially be toxic. Furthermore, these inherited disorders may result in an excess of biomaterials that do not occur in normal conditions and the body is unable to metabolize (2, 3). 

Metabolic disorders have a wide range of clinical symptoms. The prevalence of metabolic diseases varies in accordance with study population characteristics such as race, ethnic structures, or screening programs (4).

Different organs may be involved in metabolic disorders and simultaneously show pathological dysfunction.

Males and females have an equal likelihood of disease in autosomal either dominant or recessive forms. In fact, onset of disease may vary due to environmental factors and the nature of disease. Usually, newborn infants appear healthy at birth but clinical symptoms are developed later in life. More importantly, those of earlier symptoms in life, experience more acute form of the disease (5).

Metabolic disorders are commonly categorized as autosomal recessive form of metabolic disorders (6); for this reason, the history of an infant (sudden death, parental consanguinity) can be used to provide clues of the presence of such a disorder. These diseases potentially cause damage to vital organs (especially the brain) unless proper treatment is started early. Therefore, screening methods facilitating early diagnosis of metabolic diseases can preclude progressive neurological disorders and clinical manifestations that may lead to death (5).

Organic acidemia is classified into five categories as follows (7-10):

1) Branched-chain organic academia 

2) Multiple carboxylase deficiency, including holocarboxylase synthetase deficiency and biotinidase deficiency

3) Glutaric aciduria type I

4) Fatty acid oxidation defects

5) Disorders of energy metabolism

During infancy, threatening signs such as lethargy, poor feeding, and vomiting may develop because of one of these disorders; however, none of these clinical signs necessarily has to be present for an individual to have a metabolic disorder. More importantly, early diagnosis and establishment of appropriate treatment strategies can help to prevent the developmental regression and mental retardation. Paraclinical findings including metabolic acidosis, mild or moderate hyperammonemia, ketosis, sepsis-like conditions, bone marrow suppression (11-13), hypoglycemia, hepatic failure, and secondary carnitine deficiency can also help to diagnose the process (13). Diagnosis could be verified through measurement of plasma amino acids as well as the analysis of enzymes and organic acids in urine (14). Unless screening methods are employed, subclinical forms of organic acidemia might be undiagnosed.

Metabolic screening for inherited diseases is not implemented in Middle East countries; however, these diseases are more prevalent in these regions (15). 

This study aimed to evaluate some of the demographic and clinical findings in pediatric patients affected by organic acidemia.

## Materials & Methods

This cross-sectional study was part of a larger study conducted on patients with metabolic disorders during a period of 7 years (2007 to 2014) in Isfahan Province, central Iran. Our study covered a wide range of cases from newborn infants (age, one week) to 17-yr-old adolescents. Demographic information, history of disease, developmental and educational status, clinical and general conditions were obtained from hospitalized records in children’s hospitals (Imam Hussain, Azzahra, Amin) in Isfahan, Iran, known as third level referral centers and metabolic disorder centers in Isfahan Province. Information of supplementary questionnaire designed for this purpose was collected through phone and in-person interview by assistant professor of endocrinology and metabolic diseases and trained nurses.

Enzymatic and metabolic evaluation of patients’ blood was conducted at the Wagner Stibbe Laboratory in Hanover, Germany. Exclusion criteria were lack of compliance, lack of parental consent, and uncertain paraclinical features. Incidence (Incidence proportion) was estimated based on the Meikle et al., method by dividing the number of live births (n = 602,259) obtained from the Department of Census and Statistics in Isfahan Province to the number of diagnosed cases for each disease (16). According to the definition of marriage, first cousin was included cousin marriage that is marriage between people with a common grandparent.

The study was approved by the Ethical Committee of Isfahan University of Medical Sciences. Data were analyzed through a descriptive method via SPSS v22 software (IBM; Armonk, New York, USA). 

## Results

Out of 5100 patients screened in this study, 392 patients were affected by one of the metabolic disorders and 167 individuals had organic acidemia (Table 1). 

The incidence rate of organic acidemia was 27/100,000 live births (1/3606 live births in Isfahan Province). 

Propionic acidemia/methyl malonic acidemia (PA/MMA) were the most prevalent forms of metabolic disorders (1/10,383 live births). Organic acidemia was more prevalent in men (53.8%) than in women (46.2%). 

The frequency of consanguinity in patients affected by this disease was 84.7%. The absolute majority of sufferers from these diseases were inpatients (83.9%), while PA/MMA patients were more frequently hospitalized (more than 10 times as frequently according to their medical records). 42.6% cases of organic acidemia demonstrated tailored clinical signs as neonates, whereas clinical manifestations were found in most of the cases between the ages of one month and one year (50.5%). Onset of clinical signs in isovaleric acidemia and PA/MMA occurred most regularly in neonates (83.3%) and in infancy, respectively (65.5%). Furthermore, isovaleric acidemia was much more frequently diagnosed within the neonatal period (33.3%). 

**Fig 1:**
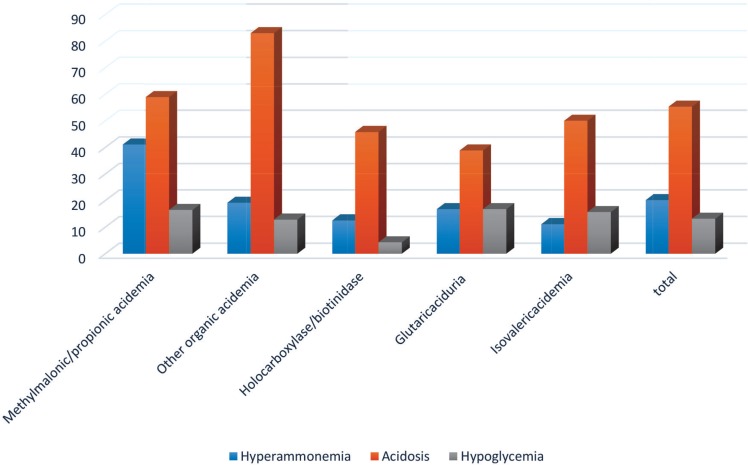
Frequency of biochemical findings in organic acidemias

Lethargy and tachypnea are the most prevalent symptoms of metabolic disorders. Most patients are affected by acidosis, and one third of them suffered from convulsions and developmental disorders. Hyperammonemia was more frequently found in PA/MMA patients (Figure 1, Table 2).

A minority of patients (9.7%) were studying in ordinary schools; the remainder were either studying in special schools for children with various disabilities or could not go to school.

A history of infant mortality was found in 12.2% of the families suffering from organic acidemia. Most of these mortalities occurred due to the lack of multiple carboxylase and biotinidase. 22.3% of mothers whose their children were affected by organic acidemia had a history of abortion. The mortality rate was 18.8% in patients with organic acidemia; one third of the population with PA/MAA died. Just about 11% of the infants were given special infant formula.

**Table 1:** Frequency of Organic Acidemia

**Percent**	**Number**	**Subtype of organic acidemia**
14.8	58	**Methylmalonic/propionic acidemia**
12.2	48	**Other organic acidemia**
6.1	24	**Holocarboxylase/biotinidase**
4.8	19	**Glutaricaciduria**
4.6	18	**Isovalericacidemia**
42.5	167	**Total**

**Table 2:** Frequency of Clinical Sign and Symptom in Organic Acidemias

**Tachypnea**	**Poor Feeding**	**Consciousness Disorder**	**Failure of Growth**	**Lethargy**	**Cardiac** **Problems**	**Developmental Delay**	**Seizure**	**Liver Dysfunction**	**Subtype Organic Acidemia**
43.1	39.6	22.4	27.5	68.9	13.7	50.9	38.2	5.5	**Methylmalonic/Propionic Acidemia**
50	20.8	14.5	22.9	70.8	14.5	17	38.3	2.1	**Other Organic Academia**
41.6	33.3	33.3	37.5	58.3	29.1	41.7	29.2	0	**Holocarboxylase/ Biotinidase**
21	21	15.7	26.3	52.6	15.7	55.6	55.6	11.1	**GlutaricAciduria**
16.6	27.7	11.1	16.6	72.2	5.5	22.2	11.1	5.6	**Isovaleric Academia**
39.5	29.9	19.7	26.3	66.4	15.5	30.8	34.4	4.9	**Total**

## Discussion

In this study, most of the metabolic disorders belonged to the organic acidemia group; two-thirds of them resulted from consanguinity. Most patients with organic academia were diagnosed before the first year, whereas in another study, half of the cases were diagnosed before the age of 3 yr (16).

In contrast to our study, this disease was rarely diagnosed in Singapore and Brazil. To elaborate, the mean age of diagnosis was during the twenty-third month in Brazilian patients; two-thirds of the patients from Singapore were between the ages of 1 and 13 yr (17, 18). 

This variety in the age of diagnosis could be ascribed to epidemiological factors (especially disease prevalence) and clinical manifestations differing in organic acidemia. 

Additionally, the ages at which patients are referred for potential diagnosis-and the frequency of timely diagnosis-may also differ according to family awareness of the potential for the disease; some families may be more aware than others of the potential for metabolic disorder in their children.

The incidence of metabolic disorders differs in many parts of the world. In our study, incidence of organic acidemia was 27/100,000 live births. These results were in accordance with similar earlier trials, where organic acidemia was more prevalent in the Middle East; however, it was less prevalent in Canada, Australia, and Italy (15, 19). To elaborate, in Italy, incidence of organic acidemia was 1/21,422 live births when PA/MMA was the most prevalent subgroup (1/61,775 live births) (16). Furthermore, we showed that incidence of organic acidemia was 1/3606 while PA/MMA incidence was 1/10,383 live births. By comparison, the incidence rate of this disease was lower in Italy (13.7%), India (41.6%), Netherlands (41.6 %), Singapore (32.3%), and Saudi Arabia (20%) and higher in China (50%) (16, 18, 20-22). Our study showed that PA/MMA were the most prevalent forms of organic acidemia, which is in accordance with analogous studies conducted in China and Saudi Arabia, Pakistan and Egypt (19, 22-25). For example, incidence rate of PA/MMA in Saudi Arabia and Karachi (Pakistan) was 29% and 62%, respectively (19, 26). Importantly, high incidence rates of organic acidemia (42%) as well as PA/MAA (23.5%) were previously proven in Shiraz, Iran (27). The possibility of metabolic diseases significantly increases when the patient shows severe clinical symptoms due to the common associated microbial infections. The patient may suffer from lack of appropriate responses to the treatment strategies. Fortunately, early diagnosis and treatment of metabolic disorders can significantly improve the disease prognosis (28); thus, developed countries are implementing selective screening that aims to detect special metabolic disorders (27). On the other hand, many of the metabolic diseases are more dangerous because they cannot be efficiently diagnosed due to the lack of patient referral to the pediatric metabolic department or to the absence of appropriate screening strategies. 

Each of the metabolic diseases, as a separate entity, is rare; nevertheless, their overall prevalence is considerable. Genotypes of these diseases generally are autosomal recessive. However, a history of sudden deaths in the family and consanguinity can be addressed as diagnostic keys for metabolic disorders. Metabolic diseases are more likely to occur when two recessive alleles of autosomal gene transfer due to consanguinity.

These data were consecutively shown in previous studies, conducted in Turkey, Saudi Arabia, Brazil and Iran (17, 29-31). For example, the rate of consanguinity marriages rate was as high as 60% in some societies, providing a background for the genetic risk factors of these metabolic diseases (30). In Turkey, Stike et al. ascribed the increase of metabolic disorders to consanguinity (29). In comparison with a previous research conducted in Iran, our study showed a higher frequency of parental consanguinity in PA/MAA patients (31).

The clinical signs attributed to organic acidemia, however, differed in accordance with the designs of different studies and the subgroups of organic acidemia.

We showed that acidosis was found in half of the cases affected by organic acidemia, while one third of these patients had some degree of developmental disorders and convulsion. In Pakistan, respiratory distress and developmental disorder were the most common clinical signs (26). In PA/MAA patients, developmental disorders, convulsion, hyperammonemia, and acidosis were demonstrated in 60%, 15%, and 25% of the cases, respectively (31). In comparison to our study, the rate of growth retardation in the aforementioned study was high (30% to 50%). Recurrent vomiting, lethargy, and respiratory distress were the most prevalent clinical signs of organic acidemia (27). In Mexico, González et al. documented different clinical signs such as vomiting, metabolic acidosis, and loss of consciousness in MAA patients (32). Organic acidemia cases showed neurologic abnormalities, metabolic acidosis, and growth retardation in 65%, 42%, and 14% of cases, respectively (17). In our study, the rate of growth retardation was higher. Nevertheless, Wajner et al. documented a high incidence rate of psychomotor retardation in organic acidemia patients, which was much more compared to our study (17).

Male/female ratio in organic acidemia patients may differ to some extent. For example, the ratios in our study and in the Brazilian study were 1:1, while in studies carried out in Libya and Pakistan the patient populations were primarily composed of men, with male: female ratios varying among the individual studies (17, 24, 33). In particular, men seemed to present to a greater degree with metabolic disorders in Libya and Pakistan. 

Our study as well as the study by Wajner et al. documented a low mortality rate (20%). On the contrary, mortality rates from metabolic disorders in France, Thailand, and China were higher (29%, 36%, and 50%, respectively); a higher mortality rate was also documented in Mexico (17, 32, 34). In a study, multiple carboxylase deficiencies had the best outcomes, and all patients treated with biotin recovered with no developmental delay, whereas mental retardation persisted in the patients with PA and MAA despite treatment. All cases with glutaric aciduria had developmental delay (23).

In general, early diagnosis of organic acidemia is essential and can potentially prevent irremediable

disorders. Neonatal screening is crucial since the disease’s outcomes are improved when the disease is diagnosed as close as possible to its onset. Nevertheless, socioeconomic problems impede the delivery of formula specialized for patients with organic acidemia; these patients are more likely to be affected by physical and mental developmental disorders. 

Lack of patient availability and cooperation, especially when they could not afford to pay for the paraclinical examinations, was assumed as our limitation. 

**In conclusion**, each of the metabolic diseases, as a separate entity, is rare; nevertheless, they are prevalent when considered as a group. These diseases could result in mental and developmental disorders without quick diagnosis and treatment, imposing costs on families and on society due to frequent inpatient treatments and the need for a specialized formula and (later) diet. 

Furthermore, more mutations should be identified in societies affected by consanguinity. However, specialized management should be applied not only to enhance life expectancy, but also to harness tardy signs of metabolic disorders. Additionally, community discussions concerning educational promotion should be sought through these types of dedicated management steps. Further research should also be conducted to determine worthwhile and more-efficient screening methods as well as long-term neurological prognosis. 
